# Leaf Age-Dependent Volatile Cues Influence Host Location and Oviposition Preference of *Obolodiplosis robiniae* on *Robinia pseudoacacia*

**DOI:** 10.3390/insects17060640

**Published:** 2026-06-17

**Authors:** Weihan Xu, Jiaqiang Zhao, Qike Wang, Zhashenjiacan Bao, Yuan Xu, Haiwei Wu, Juan Shi

**Affiliations:** 1 Beijing Key Laboratory for Forest Pest Control, Beijing Forestry University, Beijing 100083, China; xuweihan119@163.com (W.X.); baozsjc@163.com (Z.B.); xuyuan@bjfu.edu.cn (Y.X.); 2 Hebei New Area City Ecosystem Observation and Research Station, Xiongan 071800, China; 3 Shijiazhuang Institute of Fruit Trees, Hebei Academy of Agriculture and Forestry Sciences, Shijiazhuang 050061, China; jiaqiang_zhao@126.com; 4 School of BioSciences, University of Melbourne, 1 Royal Parade, Melbourne, VIC 3010, Australia; wang.q@unimelb.edu.au; 5 Shandong Academy of Forestry, Jinan 250014, China; haiweiw@126.com

**Keywords:** *Obolodiplosis robiniae*, *Robinia pseudoacacia*, tender leaves, mature leaves, oviposition preference, host location, volatile organic compounds, TD–GC–MS, Y-tube olfactometer

## Abstract

*Obolodiplosis robiniae* (Haldeman) is a specialist herbivore of *Robinia pseudoacacia* L., and its infestation is closely associated with tender leaf tissues. The ability of gravid females to recognize suitable host tissues is essential for successful oviposition and subsequent population development. Here, we assessed whether leaf age affects the host-selection behavior of *O. robiniae* and whether volatile organic compounds are associated with this process. Laboratory oviposition assays were used to compare egg deposition on tender leaves and mature leaves of *R. pseudoacacia*, and Y-tube olfactometer bioassays were performed to evaluate female responses to odors from the two leaf ages. Volatiles released from healthy tender leaves and mature leaves were collected using dynamic headspace sampling and characterized by thermal desorption–gas chromatography–mass spectrometry. Principal component analysis, orthogonal partial least squares discriminant analysis, and variable importance in projection scores were used to compare volatile profiles between leaf ages. Gravid females deposited significantly more eggs on tender leaves than on mature leaves in both choice and no-choice assays. Females also showed a significant olfactory preference for tender-leaf odors when directly offered a choice between volatiles from tender leaves and mature leaves, with 76.47% of responding individuals selecting tender-leaf odors and 23.53% selecting mature-leaf odors. Chemical profiling identified 28 volatile compounds across the two leaf ages, and their composition and relative abundance differed markedly. Among shared compounds, (Z)-3-hexen-1-ol and α-farnesene differed significantly between tender leaves and mature leaves. Multivariate analyses further identified several candidate compounds contributing to leaf age-related volatile differences. These results indicate that leaf age influences both oviposition behavior and odor-mediated host location in *O. robiniae*. Leaf age-dependent volatile blends may serve as important chemical cues associated with host selection by gravid females and provide a basis for future studies on volatile-mediated management strategies.

## Simple Summary

*Obolodiplosis robiniae* (Haldeman) is an important pest of *Robinia pseudoacacia* L., with damage mainly occurring on tender leaves. To better understand why females prefer these young tissues, we compared egg-laying behavior and odor responses of gravid females between tender leaves and mature leaves. Females laid many more eggs on tender leaves and were more attracted to odors released from tender leaves than to those from mature leaves. Chemical analysis further showed that the two leaf ages released different volatile blends. These results suggest that odors associated with leaf age may help females locate suitable oviposition sites. This study provides useful information for understanding host selection in *O. robiniae* and may support the future development of environmentally friendly control strategies based on host plant volatile cues.

## 1. Introduction

*Obolodiplosis robiniae* (Haldeman) (Diptera: Cecidomyiidae) is an invasive pest species native to eastern North America. It has spread widely across Eurasia and has become an important specialist herbivore of *Robinia pseudoacacia* L. In China, *O. robiniae* has established populations in several regions and can complete multiple generations annually under suitable climatic conditions [1]. Infestation by *O. robiniae* can cause serious damage to *R. pseudoacacia* plantations and urban forests, reducing tree vigor, ornamental value, and ecological service functions [1,2]. Females usually oviposit on newly emerged leaflets, and after hatching, the larvae feed along the leaf margins, inducing tissue proliferation and inward rolling of the leaflet margins to form characteristic galls [3]. Under severe infestation, damaged leaflets may wither and abscise prematurely, and repeated attacks can weaken shoots and reduce host plant growth. Therefore, understanding the host-selection behavior of *O. robiniae* is important for clarifying its outbreak mechanism and developing environmentally friendly management strategies [4].

Herbivorous insects and their host plants are linked through long-term ecological interactions. During host selection, insects use a range of sensory cues to detect, recognize, and evaluate suitable host plants, among which olfactory cues are particularly important [5]. Plant volatile organic compounds (VOCs) provide essential chemical information for host location, feeding-site assessment, and oviposition-site selection [6]. By detecting VOCs through olfactory organs such as antennae, herbivorous insects can distinguish host plants from non-host plants and assess host suitability before contact, thereby increasing the likelihood of successful oviposition and offspring development while reducing the risks associated with unsuitable hosts [5]. Host selection is usually a hierarchical process, involving the sequential evaluation of olfactory, visual, tactile, and contact chemical cues before final host acceptance [7].

Plant VOCs are highly dynamic and can vary with plant species, genotype, developmental stage, tissue type, physiological condition, and damage status [8]. Such variation creates distinct chemical signatures that may strongly influence insect behavioral responses [9]. In many herbivorous insects, oviposition preference is closely associated with differences in host plant volatile profiles. Specific plant volatiles may act as attractants or repellents, thereby regulating host location and oviposition behavior [10,11]. In addition, leaves at different developmental stages often differ markedly in nutritional quality, defensive chemistry, tissue structure, and volatile emissions, all of which may affect insect preference and larval performance [12,13]. Tender leaves are often more suitable for feeding and oviposition because of their softer tissues and higher nutritional value. However, for many pest species, the chemical cues underlying leaf age-dependent host preference remain poorly understood.

For *O. robiniae*, previous studies have primarily focused on its occurrence, biological characteristics, geographical distribution, and damage symptoms. However, the chemical ecological mechanisms involved in host location and oviposition-site selection remain poorly understood. In particular, whether leaf age-related differences in volatile profiles of *R. pseudoacacia* influence the host preference of gravid *O. robiniae* females has not yet been clarified. Addressing this question is important for understanding the behavioral basis of host selection in *O. robiniae* and may provide useful information for the development of semiochemical-based management strategies.

In this study, we investigated the relationship between leaf age, volatile profiles, and oviposition preference in the *R. pseudoacacia*–*O. robiniae* system. Specifically, we compared the oviposition preference of gravid *O. robiniae* females for tender leaves and mature leaves of *R. pseudoacacia* under controlled laboratory conditions. We then evaluated female behavioral responses to volatiles emitted from the two leaf ages using a Y-tube olfactometer and characterized their volatile profiles by dynamic headspace collection coupled with thermal desorption–gas chromatography–mass spectrometry (TD–GC–MS). This study aimed to determine whether leaf age-dependent volatile profiles are associated with host location and oviposition preference in *O. robiniae*. The results may improve our understanding of the potential chemical cues involved in host selection and provide a basis for future development of environmentally friendly management strategies using host plant volatiles.

## 2. Materials and Methods

### 2.1. Test Insects

**Figure 1:**
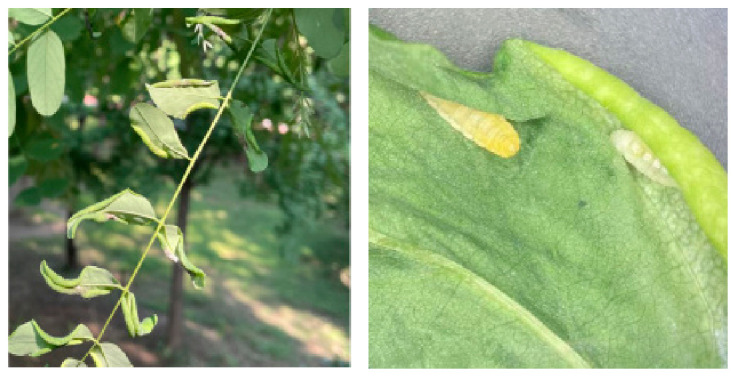
Damage symptoms caused by *O. robiniae* on host leaves.

Mature galls induced by *Obolodiplosis robiniae* (Haldeman) were collected from *Robinia pseudoacacia* L. forests at Changli Gold Coast, Qinhuangdao, Hebei Province, China (39°24′ N, 118°33′ E), and transported to the laboratory (Figure 1). The galls were placed in insect-rearing containers (10 cm in height, 5 cm in diameter) lined with fresh *R. pseudoacacia* leaves. The bases of the leaves were wrapped with moist cotton balls to maintain leaf freshness. After the larvae within the galls pupated, the galls were transferred to an artificial climate chamber (RXZ intelligent artificial climate chamber, Ningbo Jiangnan Instrument Factory, Ningbo, China) until adult emergence. The rearing conditions were maintained at 25 ± 2 °C, 55 ± 5% relative humidity, and a photoperiod of 16:8 h (L:D). Adult emergence was checked daily. Newly emerged females and males from the same cohort were transferred to insect-rearing cages (30 cm × 30 cm × 30 cm) for mating at a sex ratio of 1:1. Mating was considered complete when a male mounted a female and maintained the copulation posture for a period of time before separation. After mating, females were collected for subsequent experiments. Prior to the experiments, adult females were provided with a honey-water solution as a food source. Under laboratory conditions, this diet maintained adult survival for approximately 2–3 days, which was sufficient to complete all choice assays. Females used in the experiments had not been previously exposed to host plant odor sources, and each female was used only once.

### 2.2. Test Plants

Healthy *R*. *pseudoacacia* plants used in the experiments were obtained from the Plant Quarantine Laboratory of Beijing Forestry University, which operates under biosafety level 2 conditions. The plants were more than three years old and had not been exposed to chemical pesticides during the experimental period.

Tender leaves and mature leaves were distinguished according to their developmental stage and position on current-year shoots. Tender leaves were located at the apical part of the shoot and were light green with soft tissues, whereas mature leaves were located at the basal part of the same shoot and were dark green with firmer tissues.

### 2.3. Oviposition Preference of O. robiniae for Tender Leaves and Mature Leaves of R. pseudoacacia

The oviposition preference of gravid *O. robiniae* females for tender leaves and mature leaves of *R. pseudoacacia* was evaluated using both choice and no-choice assays, following the method of Zhang et al. [14] with slight modifications. All assays were conducted under controlled conditions of 25 ± 2 °C, 35 ± 5% relative humidity, and a photoperiod of 16:8 h (L:D).

In the choice assay, *R. pseudoacacia* shoots bearing either tender leaves or mature leaves were used as oviposition substrates. Each shoot contained 17 leaves, and three shoots were prepared for each leaf age. The shoots were inserted separately into glass bottles filled with distilled water (5.6 cm in diameter and 12 cm in height) to maintain leaf freshness. Glass bottles containing shoots of the two leaf ages were fixed along the diagonal of an insect-rearing cage (30 cm × 30 cm × 30 cm), allowing females to contact both tender leaves and mature leaves simultaneously. Twenty mated gravid *O. robiniae* females were then released into each cage and allowed to move freely. A Petri dish containing cotton balls soaked with honey solution was placed in the center of the cage as a nutrient source. Beginning 24 h after female release, cages were inspected daily at 09:00, 13:00, and 17:00 to monitor female survival and leaf condition. Under laboratory conditions, females provided with honey solution generally survived for 2–3 days. The experiment continued until all test females had died, after which all shoots were collected and the number of eggs deposited on each leaf was counted under a stereomicroscope. The choice assay was replicated three times, with each cage considered one replicate.

In the no-choice assay, shoots bearing either tender leaves or mature leaves of *R. pseudoacacia* were fixed separately in the center of independent insect-rearing cages (30 cm × 30 cm × 30 cm). Twenty mated gravid *O. robiniae* females were released into each cage, and the cage was then closed to prevent insect escape. A Petri dish containing cotton balls soaked with honey solution was also placed in each cage to maintain adult survival. Oviposition behavior was observed as described above. After all females had died, the shoots were removed, and the number of eggs deposited on the leaves was counted under a stereomicroscope. The no-choice assay was replicated three times for each leaf age, with each cage considered one replicate.

The oviposition selection rate and mean number of eggs per leaf were calculated to quantify female oviposition preference. In the choice assay, oviposition selection rate was calculated as:

$$Oviposition\;selection\;rate\left(\%\right)=\frac{Total\;number\;of\;eggs\;on\;one\;host\;plant\;type}{Total\;number\;of\;eggs\;on\;both\;host\;plant\;types}\times 100$$

The mean number of eggs per leaf was calculated as:

$$Mean\;number\;of\;eggs\;per\;leaf=\frac{Total\;number\;of\;eggs}{Total\;number\;of\;leaves}$$

In the no-choice assay, the mean number of eggs per leaf was calculated using the same method.

### 2.4. Behavioral Responses of O. robiniae Females to Volatiles from Tender Leaves and Mature Leaves

The behavioral responses of female *O. robiniae* to volatiles emitted from tender leaves and mature leaves of *R. pseudoacacia* were tested using a Y-tube olfactometer. Three odor-source combinations were tested: (1) tender leaves versus blank control; (2) mature leaves versus blank control; and (3) tender leaves versus mature leaves.

Bioassays were conducted between 08:00 and 16:00 under controlled conditions of 25 ± 2 °C, 35 ± 5% relative humidity, and 240 lx illumination. Airflow was generated using an atmospheric sampling pump. Incoming air was purified through activated charcoal and humidified with distilled water before entering the Y-tube olfactometer. The Y-tube consisted of a 25 cm main arm and two 15 cm side arms, with an angle of 60° between the side arms. The airflow rate was maintained at 120 mL·min^−1^.

For each test, a single female *O. robiniae* was introduced into the main arm of the Y-tube. The insect was allowed to move freely within the olfactometer. Timing began when the female reached the bifurcation point (i.e., the junction where the main arm divided into the two side arms and where the female was simultaneously exposed to the two odor streams). A choice was recorded when the female moved more than two-thirds of the distance into one side arm within 5 min and remained there for at least 1 min. Females that did not make a clear choice within 5 min were recorded as non-responders. Non-responders were recorded but not included in the comparison of choices between odor sources. Twenty females were tested for each odor-source combination. After every five females, the positions of the two odor-source bottles and the orientation of the Y-tube were exchanged to minimize positional bias. After each test, the entire olfactometer system was cleaned with anhydrous ethanol and air-dried before reuse.

### 2.5. Collection of Volatiles from Tender Leaves and Mature Leaves of R. pseudoacacia

Volatiles released from tender leaves and mature leaves of *R. pseudoacacia* were collected in vivo using a closed-loop dynamic headspace sampling. Tender-leaf shoots and mature-leaf shoots were selected from the same healthy *R. pseudoacacia* plant. Four shoots were selected for each leaf age.

The volatile collection system was assembled following Wang et al. [15] with slight modifications. Before sampling, all devices were thoroughly cleaned and dried in an oven. During sampling, each shoot was enclosed in an EasyOven oven bag (43 cm × 55 cm) to form a headspace collection chamber. Rigid polytetrafluoroethylene tubes, with an inner diameter of 1 cm and a length of 20 cm, were inserted into the upper and lower ends of the bag. The upper tube was connected to a CAMSCO (Taipei, Taiwan) stainless-steel thermal desorption tube packed with Tenax GR 60/80, which was used to adsorb and trap plant-emitted volatiles. The lower tube was connected to an atmospheric sampling pump (QC-1B, Beijing Municipal Institute of Labour Protection, Beijing, China) The activated-charcoal filter bottle was used to remove background contaminants from the incoming air, whereas the QC-1B served as the airflow-driving device. Together, these components formed a closed-loop dynamic headspace collection system. All connections were sealed with parafilm to prevent air leakage. Continuous airflow was maintained throughout the sampling period to minimize moisture accumulation within the collection system, and no visible condensation was observed on the adsorbent tube during volatile collection. Air samples collected from empty bags were used as blank controls to eliminate interference from environmental background contaminants. Volatile collection was conducted from 08:00 to 16:00 for 8 h. Each tree was considered one biological replicate, and three biological replicates were performed.

### 2.6. Analysis of Volatile Compounds

Leaf volatiles were analyzed using a Turbo Matrix 350 automatic thermal desorber coupled to a Clarus 690 gas chromatograph and a Clarus SQ 8T mass spectrometer (PerkinElmer, Waltham, MA, USA). Volatiles were desorbed using a two-stage thermal desorption procedure. The primary desorption temperature was set at 260 °C for 10 min, and the cold trap temperature was maintained at −30 °C. Secondary desorption was performed at 300 °C for 5 min, with a cold-trap heating rate of 40 °C/s. The sample introduction ratio was set at 4%. Gas chromatographic separation was performed on an Elite-5MS capillary column (30 m × 0.25 mm × 0.25 μm). Helium was used as the carrier gas at a flow rate of 1.5 mL/min. The injector temperature was set at 250 °C, and samples were introduced in splitless mode. The oven temperature program was as follows: the initial temperature was held at 40 °C for 2 min, increased to 180 °C at 6 °C/min, then increased to 270 °C at 20 °C/min and held for 3 min. Mass spectrometric detection was performed in electron ionization (EI) mode at 70 eV. The detector temperature was 250 °C, the ion source temperature was 230 °C, and the mass scan range was set from m/z 29 to 500.

### 2.7. Determination of Retention Indices

Retention indices were calculated to verify the reliability of compound identification. A C10–C25 n-alkane standard mixture (Aladdin Biochemical Technology Co., Ltd., Shanghai, China) was analyzed under the same GC–MS conditions described above. The retention index (KI) of each compound was calculated using the following equation:

$$KI=100n+\frac{100(t_{x}-t_{n})}{100(t_{n+1}-t_{n})}$$

where *t_x_*, *t_n_*, *t_n_*_+1_, represent the retention times of the target compound, the n-alkane eluting immediately before the target compound, and the n + 1 alkane eluting immediately after the target compound, respectively.

### 2.8. Statistical Analysis

All statistical analyses were performed using SIMCA 14.1 and SPSS 27.0. Oviposition preference data were analyzed using independent-samples *t*-tests. The oviposition selection rate was arcsine square-root transformed before analysis, and the mean number of eggs per leaf was compared between tender leaves and mature leaves. Behavioral response data from the Y-tube olfactometer assays were analyzed using the chi-square test. For volatile analysis, background signals from blank air samples were subtracted before further processing. Volatile compounds were qualitatively identified by combining NIST database matching with manual correction of characteristic peaks. Relative contents of compounds were calculated using the peak area normalization method. Principal component analysis (PCA) and orthogonal partial least squares discriminant analysis (OPLS–DA) models were constructed to compare volatile profiles between tender leaves and mature leaves. Model stability was evaluated using permutation tests. Differential volatile compounds were screened using the criteria of variable importance in projection (VIP) > 1 and *p* < 0.05. Figures were generated using GraphPad Prism 10.1.2.

## 3. Results

### 3.1. Oviposition Preference of O. robiniae Females for Leaves of Different Ages

**Figure 2:**
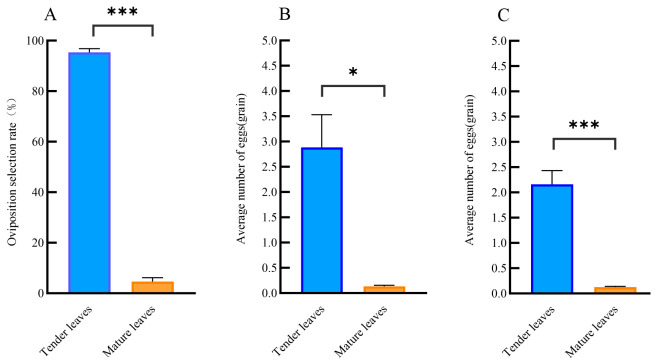
Oviposition preference of gravid *O. robiniae* females for tender and mature leaves of *R. pseudoacacia*. (**A**) Oviposition selection rate in the choice assay; (**B**) mean number of eggs per leaf in the choice assay; (**C**) mean number of eggs per leaf in the no-choice assay. Statistical significance is indicated as * *p* < 0.05, *** *p* < 0.001 (*t*-test).

Under choice conditions, gravid *O. robiniae* females showed a strong preference for oviposition on tender leaves. The oviposition selection rate for tender leaves was 95.33%, which was significantly higher than that for mature leaves, 4.67% (*p* < 0.001) (Figure 2A). The mean number of eggs deposited per leaf was also significantly higher on tender leaves than on mature leaves (*p* < 0.05) (Figure 2B). On average, approximately three eggs were deposited per tender leaf, whereas fewer than one egg was deposited per mature leaf.

In the no-choice assay, females deposited significantly more eggs on tender leaves than on mature leaves. Eggs deposited in the tender-leaf treatment accounted for 91.55% of the total eggs recorded in the no-choice assay, whereas those in the mature-leaf treatment accounted for 8.45% (*p* < 0.001) (Figure 2C). Egg counts refer to individual eggs rather than egg masses, and all eggs were counted separately under a stereomicroscope. These results indicate that *O. robiniae* females exhibit a clear preference for tender leaves during oviposition, while mature leaves are rarely selected.

### 3.2. Behavioral Responses of Gravid O. robiniae Females to Volatiles from Tender Leaves and Mature Leaves

**Figure 3:**
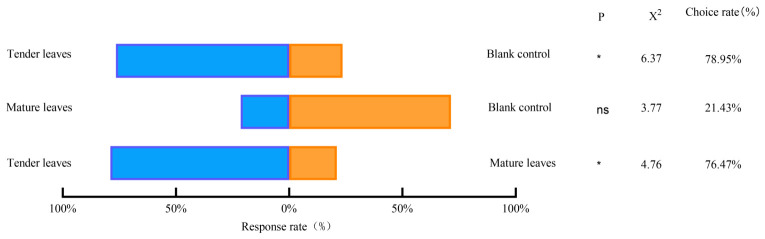
Behavioral responses of gravid *O. robiniae* females to volatiles from tender and mature leaves of *R. pseudoacacia* in Y-tube olfactometer bioassays. Bars of different colors represent the responses to different odor sources. “*” indicates a significant difference between odor sources based on chi-square tests (*p* < 0.05), whereas “ns” indicates no significant difference.

In the Y-tube olfactometer bioassays, gravid *O. robiniae* females showed different behavioral responses to volatiles emitted from tender leaves and mature leaves of *R. pseudoacacia* (Figure 3). When tender-leaf volatiles were tested against the blank control, females showed a significant positive response to tender-leaf odors, with a response rate of 78.95% (χ^2^ = 6.37, df = 1, *p* < 0.05). In contrast, when mature-leaf volatiles were tested against the blank control, females showed no significant attraction or avoidance of mature-leaf odors, with a response rate of 21.43% (χ^2^ = 3.77, df = 1, *p* > 0.05). When volatiles from tender leaves and mature leaves were directly compared, tender-leaf odors were significantly more attractive to gravid females than mature-leaf odors, with response rates of 76.47% and 23.53%, respectively (χ^2^ = 4.76, df = 1, *p* < 0.05).

### 3.3. Volatile Profiles of Tender Leaves and Mature Leaves of R. pseudoacacia

**Figure 4:**
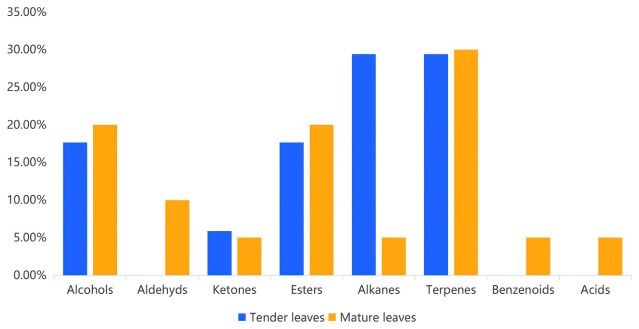
Differences in volatile compound classes detected from tender leaves and mature leaves of *R. pseudoacacia*. Blue represents tender leaves, and orange represents mature leaves.

The volatile profiles of tender leaves and mature leaves of *R. pseudoacacia* differed in compound composition and relative abundance. A total of 28 volatile compounds were detected across the two leaf ages, mainly including alcohols, aldehydes, ketones, esters, alkanes, terpenoids, benzenoids, and organic acids (Table 1). Seventeen compounds were detected from tender leaves. Among these, terpenoids and alkanes were the dominant chemical classes, with five compounds each, accounting for 29.41% of the total number of volatile compounds, respectively. In addition, three esters, three alcohols, and one ketone were detected in tender leaves (Figure 4).

Twenty compounds were detected from mature leaves. These included six terpenoids, four alcohols, four esters, two aldehydes, one ketone, one benzenoid, and one organic acid (Figure 4). As shown in Table 1, eight compounds were detected only in tender leaves: 2-octyl-1-dodecanol, isophorone, pentadecane, heneicosane, tetracosane, pentacosane, 1,4-pentadiene, and 1,3,5,5-tetramethyl-1,3-cyclohexadiene. In contrast, eleven compounds were detected only in mature leaves: 2-hexyn-1-ol, tert-hexadecyl mercaptan, (E)-2-pentenal, benzaldehyde, 6,10-dimethyl-5,9-undecadien-2-one, hexyl acetate, (+)-α-pinene, (E)-4,8-dimethyl-1,3,7-nonatriene, β-caryophyllene, *p*-xylene, and acetic acid.

Among the compounds detected in both leaf ages, the relative abundances of (Z)-3-hexen-1-ol and α-farnesene differed significantly between tender leaves and mature leaves (*p* < 0.01 and *p* < 0.05, respectively). These results indicate that leaf age markedly affects the volatile composition of *R. pseudoacacia*.

### 3.4. PCA and OPLS–DA Analyses of Volatiles from Tender Leaves and Mature Leaves of R. pseudoacacia

**Figure 5:**
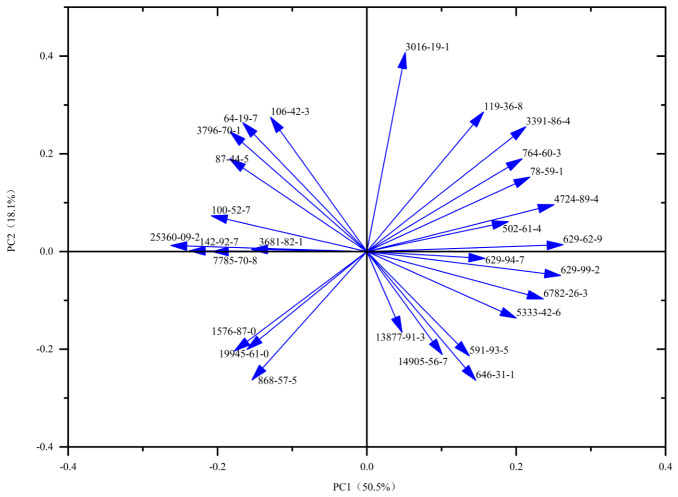
Principal component analysis (PCA) of volatile compounds from tender leaves and mature leaves of *R. pseudoacacia*.

Principal component analysis (PCA) was performed to evaluate differences in volatile profiles between tender leaves and mature leaves of *R. pseudoacacia* (Figure 5). The first principal component (PC1) explained 50.5% of the total variance, whereas the second principal component (PC2) explained 18.1%. Together, PC1 and PC2 accounted for 68.6% of the total variance. The PCA score plot showed a separation trend between tender-leaf and mature-leaf samples along PC1. The PCA loading results indicated that compounds such as α-farnesene, benzaldehyde, and β-caryophyllene contributed to the differentiation of volatile profiles between the two leaf ages.

**Figure 6:**
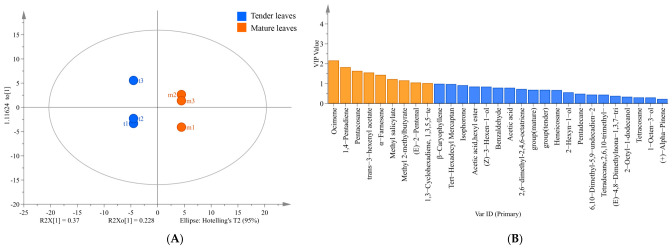
OPLS–DA analysis of volatile compounds from tender leaves and mature leaves of *R. pseudoacacia*. (**A**) Score plot; (**B**) variable importance in projection (VIP) scores.

OPLS–DA analysis further supported the separation between tender-leaf and mature-leaf samples (Figure 6A). Samples from the same leaf age clustered closely, indicating good repeatability within groups. Because of the limited number of biological replicates, the OPLS–DA model was used as an exploratory approach to identify candidate compounds contributing to volatile differences between leaf ages. Based on VIP scores, nine volatile compounds with VIP values > 1 were identified as important contributors to the differences between tender leaves and mature leaves (Figure 6B).

## 4. Discussion

Host selection and oviposition-site selection are key behavioral processes that affect the establishment and population development of herbivorous insects. For many phytophagous insects, adult females determine the developmental environment of their offspring through oviposition decisions, especially when larvae have limited mobility after hatching [5,16]. In the present study, gravid *O. robiniae* females showed a clear preference for tender leaves of *R. pseudoacacia* in both choice and no-choice assays. Although eggs were also deposited on mature leaves, both the oviposition selection rate and the mean number of eggs per leaf were significantly higher on tender leaves. This result is consistent with field observations and previous reports showing that *O. robiniae* females mainly lay eggs on newly emerged leaf tissues and that larval feeding induces inward rolling of leaflet margins and gall formation [17,18]. Therefore, the preference for tender leaves appears to be closely associated with the natural damage pattern and biological characteristics of *O. robiniae*.

The preference of gravid females for tender leaves may reflect an adaptive strategy that increases offspring survival and performance. According to the preference–performance hypothesis, female herbivores are expected to prefer host tissues or host plants on which their offspring can achieve better growth and survival [19]. This relationship may be particularly important for gall-inducing insects, because successful gall formation depends not only on host identity but also on the developmental and physiological status of the plant tissue. Tender leaves generally have softer tissues, higher water content, more active cell division, and weaker mechanical defenses than mature leaves. These properties may facilitate larval feeding, tissue manipulation, and the formation of gall structures. Gall-inducing insects are known to manipulate plant developmental processes and induce specialized plant tissues that support larval development [20,21]. Thus, the strong oviposition preference of *O. robiniae* for tender leaves may represent a behavioral adaptation to select tissues that are more suitable for larval establishment and gall development. However, the present study did not evaluate egg hatchability, gall formation success, or larval developmental performance on leaves of different ages. Therefore, whether offspring achieve higher fitness on tender leaves remains to be determined and requires further investigation.

The Y-tube olfactometer assays further demonstrated that leaf volatiles are involved in host location by gravid *O. robiniae* females. Females showed significant attraction to volatiles from tender leaves when tested against the blank control, whereas the response to mature-leaf volatiles was relatively weak. When volatiles from tender leaves and mature leaves were directly compared, females significantly preferred tender-leaf odors. Together, these results indicate that gravid *O. robiniae* females preferentially respond to volatiles emitted from tender leaves. These results indicate that leaf age-dependent odor cues may help gravid females identify suitable oviposition sites before physical contact with the host tissue. Plant volatile organic compounds are known to play important roles in host location by herbivorous insects, and insects may use either species-specific compounds or characteristic ratios of common compounds to recognize host plants [22]. Therefore, the attraction of *O. robiniae* females to tender-leaf volatiles suggests that chemical cues associated with leaf developmental stage contribute to host recognition in this system.

However, host selection by herbivorous insects is usually a sequential and multimodal process. Volatile cues may guide insects toward suitable host tissues from a distance, but final host acceptance often depends on additional cues, including leaf color, shape, surface structure, texture, and contact chemical signals [8]. In the present study, the olfactometer results confirm that tender-leaf odors are attractive to gravid females, but they do not exclude the possible contribution of non-volatile traits. The higher oviposition on tender leaves observed in the choice and no-choice assays may therefore result from the combined effects of leaf volatiles and contact-based indicators of host quality. Future studies that separate olfactory, visual, and contact cues would help clarify the relative contribution of each cue type to host selection by *O. robiniae*.

Chemical analysis showed that tender leaves and mature leaves of *R. pseudoacacia* differed clearly in both volatile composition and relative abundance. A total of 28 volatile compounds were detected across the two leaf ages, mainly including alcohols, esters, alkanes, terpenoids, aldehydes, ketones, benzenoids, and organic acids. Tender leaves were characterized by relatively high proportions of terpenoids and alkanes, whereas mature leaves contained relatively high proportions of terpenoids, esters, and alcohols. These results suggest that leaf developmental stage strongly influences the volatile profile of *R. pseudoacacia*. Similar effects of leaf age on volatile composition and arthropod behavioral responses have been reported in other plant–arthropod systems [23]. Such age-related variation in plant volatile emissions is ecologically important because insects can use differences in volatile blends to evaluate host identity, host quality, and tissue suitability [24]. The attraction of gravid *O. robiniae* females to tender-leaf odors observed in this study is therefore likely associated with the distinct volatile blend emitted by tender leaves. Among the compounds detected in both leaf ages, the relative abundances of (Z)-3-hexen-1-ol and α-farnesene differed significantly. Green leaf volatiles, including (Z)-3-hexen-1-ol and related C6 compounds, are widely involved in plant–insect interactions and can influence host searching, feeding, and oviposition behavior [25,26]. α-Farnesene is also a common plant volatile and has been reported as an attractant and oviposition stimulant in some insect–host plant systems [27]. Therefore, the significant differences in (Z)-3-hexen-1-ol and α-farnesene between tender leaves and mature leaves may contribute to leaf age-specific odor signatures in *R. pseudoacacia*. Nevertheless, the behavioral function of a compound cannot be inferred solely from its presence or relative abundance. Insect responses to plant volatiles often depend on compound concentration, physiological state of the insect, and the ratio of compounds within the whole volatile blend [28]. Thus, these compounds should be regarded as candidate cues associated with leaf age-dependent host selection, and their specific roles in *O. robiniae* require further behavioral and electrophysiological validation.

It should be noted that the present study focused primarily on differences in volatile composition and relative abundance between tender and mature leaves. Absolute emission rates of volatile compounds were not quantified; therefore, the potential contribution of quantitative differences in volatile emissions to the observed behavioral responses cannot be completely excluded. In addition to qualitative differences in volatile blends, variation in emission intensity may also influence insect attraction and host-selection behavior. Future studies incorporating quantitative analyses of volatile emissions will help clarify the relative importance of volatile composition and dose-dependent effects in host selection by *O. robiniae*.

The PCA and OPLS–DA analyses further supported the differentiation of volatile profiles between tender leaves and mature leaves. PCA showed a separation trend between the two leaf ages, while OPLS–DA and VIP analysis identified several compounds that contributed strongly to group separation, including ocimene, 1,4-pentadiene, *n*-pentacosane, (E)-3-hexen-1-ol acetate, α-farnesene, methyl salicylate, methyl 2-methylbutanoate, trans-2-pentenal, and 1,3,5,5-tetramethyl-1,3-cyclohexadiene. These compounds may be regarded as candidate volatiles associated with leaf age-related differences. However, compounds identified by multivariate analyses are not necessarily the compounds that directly mediate insect behavioral responses. Insect responses to plant volatiles are often determined by the overall quality and ratio of the volatile blend rather than by a single dominant compound [7,25]. Therefore, the stronger response of *O. robiniae* females to tender-leaf odors may reflect their perception of the entire tender-leaf volatile blend. Some compounds may have weak or no behavioral activity when tested alone but may enhance or suppress behavioral responses when combined with other volatiles [29]. Thus, these candidate volatiles should be interpreted as targets for further behavioral and electrophysiological validation rather than as confirmed active cues.

Several compounds identified in this study have been reported to influence insect behavior in other systems. For example, methyl salicylate is a well-known plant volatile involved in plant defense signaling and has been widely investigated for its ecological effects on insects and natural enemies [30]. However, methyl salicylate may also function as an oviposition deterrent in some plant–insect systems, indicating that its ecological role is context-dependent [31]. β-Caryophyllene is a common sesquiterpene involved in plant–insect communication, although its ecological function may vary depending on insect species and concentration [32]. Isophorone, esters, and aldehydes may also contribute to host odor profiles. However, because insect responses are species-specific and context-dependent, the functions of these compounds in the *R. pseudoacacia*–*O. robiniae* system cannot be inferred solely from studies on other insects or from their relative abundance in plant volatile profiles [29]. Their specific roles should be confirmed through single-compound bioassays, synthetic blend tests, and electrophysiological analyses.

Overall, this study provides behavioral and chemical evidence that leaf age affects host selection and oviposition by *O. robiniae*. Gravid females preferentially deposited eggs on tender leaves and were significantly attracted to tender-leaf volatiles, while tender leaves and mature leaves differed markedly in volatile composition. These findings suggest that leaf age-dependent volatile blends may provide important chemical information during host location and oviposition-site selection. Nevertheless, this study has several limitations. First, individual compounds and synthetic blends were not tested, so the specific active compounds responsible for female attraction remain unknown. Second, antennal responses of *O. robiniae* to candidate compounds were not measured. Third, laboratory olfactometer responses may not fully represent insect behavior under field conditions, where background odors, wind movement, host density, and plant phenology may influence host searching. Background odors are known to modify insect responses to resource-indicating cues, and thus semi-field or field validation is necessary before these candidate compounds can be applied in management strategies [33]. Future studies should test candidate compounds individually and in mixtures at ecologically relevant concentrations, combine behavioral assays with electroantennography or gas chromatography–electroantennographic detection, and evaluate their effects under semi-field or field conditions. Such work would further clarify the chemical ecological mechanisms underlying host selection in *O. robiniae* and may support the development of volatile-based monitoring or management strategies for this pest.

## 5. Conclusions

This study demonstrated that gravid *O. robiniae* females show a significant oviposition preference for tender leaves of *R. pseudoacacia* and are more strongly attracted to tender-leaf volatiles than to mature-leaf volatiles. Tender leaves and mature leaves differed in volatile composition and relative abundance. Terpenoids and alkanes were dominant in tender leaves, whereas terpenoids and esters were relatively abundant in mature leaves. Multivariate analyses indicated that several compounds, including ocimene, 1,4-pentadiene, *n*-pentacosane, (E)-3-hexen-1-ol acetate, and α-farnesene, contributed to the differentiation of volatile profiles between leaf ages. These results suggest that leaf age-dependent volatile blends are associated with host location and oviposition-site selection in *O. robiniae*. The candidate compounds identified here provide targets for further behavioral and electrophysiological studies, but their specific functions require experimental verification.

## Figures and Tables

**Table 1:** Volatile compounds detected from tender leaves and mature leaves of *R. pseudoacacia*.

No.	Retention Time (min)	Compounds	KI	CAS No.	Relative Content (%)	
					Tender Leaves	Mature Leaves
		Alcohols				
1	5.826	2-Hexyn-1-ol	847	764-60-3	–	0.74 ± 0.260
2	6.086	(Z)-3-Hexen-1-ol	857	6782-26-3	1.94 ± 0.137 *	0.38 ± 0.033 *
3	24.289	2-Octyl-1-dodecanol	1079	5333-42-6	0.26 ± 0.092	–
4	24.303	1-Octen-3-ol	1395	3391-86-4	0.78 ± 0.235	0.57 ± 0.267
5	26.030	tert-Hexadecyl mercaptan	1844	25360-09-2	–	2.26 ± 1.064
		Aldehydes				
6	3.76	(E)-2-Pentenal	754	1576-87-0	–	3.19 ± 0.798
7	8.702	Benzaldehyde	961	100-52-7	–	1.59 ± 0.691
		Ketones				
8	12.849	Isophorone	1117	78-59-1	1.94 ± 0.271	–
9	20.307	6,10-Dimethyl-5,9-undecadien-2-one	1436	3796-70-1	–	0.53 ± 0.250
		Esters				
10	4.36	Methyl 2-methylbutyrate	777	868-57-5	2.93 ± 0.601	6.44 ± 0.895
11	9.888	(E)-3-Hexenyl acetate	1004	3681-82-1	5.9 ± 0.755	11.55 ± 1.744
12	10.058	Hexyl acetate	1036	142-92-7	–	1.59 ± 0.647
13	14.615	Methyl salicylate	1190	119-36-8	4.91 ± 0.901	1.33 ± 0.438
		Alkanes				
14	21.459	Pentadecane	1500	629-62-9	0.51 ± 0.181	–
15	24.304	2,6,10-Trimethyltetradecane	1555	14905-56-7	1.44 ± 0.063	0.53 ± 0.248
16	29.567	Heneicosane	2100	629-94-7	1.08 ± 0.284	–
17	30.257	Tetracosane	2400	646-31-1	0.22 ± 0.077	–
18	31.483	Pentacosane	2500	629-99-2	5.72 ± 1.892	–
		Terpenes				
19	1.844	1,4-Pentadiene	480	591-93-5	7.97 ± 1.764	–
20	8.012	(+)-α-Pinene	939	7785-70-8	–	0.12 ± 0.058
21	10.948	Ocimene	1041	13877-91-3	43.09 ± 2.523	34.9 ± 5.472
22	12.684	(E)-4,8-Dimethyl-1,3,7-nonatriene	1110	19945-61-0	–	0.38 ± 0.178
23	12.999	1,3,5,5-tetramethyl-1,3-cyclohexadiene	1020	4724-89-4	2.97 ± 1.050	–
24	13.039	2,6-dimethyl-2,4,6-octatriene	1128	3016-19-1	3.44 ± 0.531	3.21 ± 1.513
25	19.817	β-Caryophyllene	1614	87-44-5	–	4.57 ± 0.642
26	21.508	α-Farnesene	1508	502-61-4	8.55 ± 1.166 *	2.97 ± 0.748 *
		Benzenoids				
27	6.381	*p*-xylene	883	106-42-3	–	0.56 ± 0.264
		Acids				
28	2.494	Acetic acid	600	64-19-7	–	1.81 ± 0.505

Note: Data are presented as mean ± SE. Asterisks indicate significant differences between tender leaves and mature leaves (*p* < 0.05). “–” indicates not detected. KI, retention index. Compounds were putatively identified based on mass spectral matching and retention indices where applicable.

## Data Availability

The data presented in this study are available on request from the corresponding author.

## References

[1] Zhao J.Q., Gao T., Du J.J., Shi J. (2023). Future trends in *Obolodiplosis robiniae* distribution across Eurasian continent under global climate change. Insects.

[2] Huang L., Wang L., Sun H.Q., Huai W.X., Lin R.Z., Wei S.J., Yao Y.X. (2024). The chromosome-level genome assembly and annotation of an invasive forest pest *Obolodiplosis robiniae*. Sci. Data.

[3] Ilea I., Hulujan I.B., Florian T., Florian V., Oltean I. (2023). The gall midge *Obolodiplosis robiniae* Haldemann (Diptera Cecidomyiidae) new invasive alien species in Europa-Review. Not. Bot. Horti Agrobot. Cluj-Napoca.

[4] Shang X.P., Yao Y.X., Zhao W.X. (2015). Geographic distribution of an invasive insect pest, *Obolodiplosis robiniae* (Diptera: Cecidomyiidae) in China. For. Pest Dis..

[5] Gripenberg S., Mayhew P.J., Parnell M., Roslin T. (2010). A meta-analysis of preference–performance relationships in phytophagous insects. Ecol. Lett..

[6] Jeffrey K.B. (1993). Biology of Insect-Induced Galls. Ann. Entomol. Soc. Am..

[7] Bruce T.J.A., Wadhams L.J., Woodcock C.M. (2005). Insect host location: A volatile situation. Trends Plant Sci..

[8] Carrasco D., Larsson M.C., Anderson P. (2015). Insect host plant selection in complex environments. Curr. Opin. Insect Sci..

[9] Anton S., Cortesero A. (2022). Plasticity in chemical host plant recognition in herbivorous insects and its implication for pest control. Biology.

[10] Unsicker S.B., Kunert G., Gershenzon J. (2009). Protective perfumes: The role of vegetative volatiles in plant defense against herbivores. Curr. Opin. Plant Biol..

[11] Dicke M., Baldwin I.T. (2010). The evolutionary context for herbivore-induced plant volatiles: Beyond the ‘cry for help’. Trends Plant Sci..

[12] Snoeren T.A.L., Mumm R., Poelman E.H., Yang Y., Pichersky E., Dicke M. (2010). The herbivore-induced plant volatile methyl salicylate negatively affects attraction of the parasitoid *Diadegma semiclausum*. J. Chem. Ecol..

[13] Lee J.C. (2022). Methyl salicylate can benefit ornamental pest control by increasing natural enemy abundance. Front. Ecol. Evol..

[14] Zhang Y.Q., Sun H.Y., Feng J.K., Xu C.Q., Liu S., Guo K., Wei H.S., Xu R., Qiao H.L., Lu P.F. (2023). Oviposition preferences of *Gephyraulus lycantha* (Diptera: Cecidomyiidae) females with respect to three *Lycium* spp. host plants. Chin. J. Appl. Entomol..

[15] Wang M.X., Zhang X.W., Ren G.J., Wang J., Li Y., Qu Y.Y., Ma A.B., Shi L., Duan B.N., Wu H.W. (2025). Analysis of secondary metabolic phenols and volatile compounds insix host plants of *Matsucoccus matsumurae*. Chin. J. Appl. Entomol..

[16] Mayhew P.J. (1997). Adaptive patterns of host-plant selection by phytophagous insects. Oikos.

[17] Pei C.Y., Zheng C.Y. (2011). Selectivity of *Frankliniella occidentalis* (Pergande) on different host vegetables. Chin. J. Eco-Agric..

[18] Zhang D.F., Lu C.K., Wang X.Q., Gao B.J. (2009). Potential risk assessment of *Obolodiplosis robiniae* (Haldemann) in China. Acta Ecol. Sin..

[19] Thompson J.N. (1988). Evolutionary ecology of the relationship between oviposition preference and performance of offspring in phytophagous insects. Entomol. Exp. ET Appl..

[20] Oliveira D.C., Isaias R.M.S., Fernandes G.W., Ferreira B.G., Carneiro R.G.S., Fuzaro L. (2016). Manipulation of host plant cells and tissues by gall-inducing insects and adaptive strategies used by different feeding guilds. J. Insect Physiol..

[21] Stone G.N., Schönrogge K. (2003). The adaptive significance of insect gall morphology. Trends Ecol. Evol..

[22] Visser J.H. (1986). Host odor perception in phytophagous insects. Annu. Rev. Entomol..

[23] Takabayashi J., Dicke M., Takahashi S., Posthumus M.A., van Beek T.A. (1994). Leaf age affects composition of herbivore-induced synomones and attraction of predatory mites. J. Chem. Ecol..

[24] Bruce T.J.A., Midega C.A.O., Birkett M.A., Pickett J.A., Khan Z.R. (2011). Perception of plant volatile blends by herbivorous insects—Finding the right mix. Phytochemistry.

[25] Scala A., Allmann S., Mirabella R., Haring M.A., Schuurink R.C. (2013). Green leaf volatiles: A plant’s multifunctional weapon against herbivores and Pathogens. Int. J. Mol. Sci..

[26] Wei J., Kang L. (2011). Roles of (Z)-3-hexenol in plant-insect interactions. Plant Signal. Behav..

[27] Sutherland O.R.W., Wearing C.H., Hutchins R.F.N. (1977). Production of α-farnesene, an attractant and oviposition stimulant for codling moth, by developing fruit of ten varieties of apple. J. Chem. Ecol..

[28] Szendrei Z., Rodriguez-Saona C. (2010). A meta-analysis of insect pest behavioral manipulation with plant volatiles. Entomol. Exp. Appl..

[29] van Wijk M., de Bruijn P.J.A., Sabelis M.W. (2008). Predatory mite attraction to herbivore-induced plant odors is not a consequence of attraction to individual herbivore-induced plant volatiles. J. Chem. Ecol..

[30] Salamanca J., Souza B., Kyryczenko-Roth V., Rodriguez-Saona C. (2019). Methyl salicylate increases attraction and function of beneficial arthropods in cranberries. Insects.

[31] Groux R., Hilfiker O., Gouhier-Darimont C., Peñaflor M.F.G.V., Erb M., Reymond P. (2014). Role of methyl salicylate on oviposition deterrence in *Arabidopsis thaliana*. J. Chem. Ecol..

[32] Riddick E.W. (2020). Volatile and Non-Volatile Organic Compounds Stimulate Oviposition by Aphidophagous Predators. Insects.

[33] Schröder R., Hilker M. (2008). The relevance of background odor in resource location by insects: A behavioral approach. BioScience.

